# Astrocyte TNFR2 is required for CXCL12-mediated regulation of oligodendrocyte progenitor proliferation and differentiation within the adult CNS

**DOI:** 10.1007/s00401-012-1034-0

**Published:** 2012-08-30

**Authors:** Jigisha R. Patel, Jessica L. Williams, Megan M. Muccigrosso, Laindy Liu, Tao Sun, Joshua B. Rubin, Robyn S. Klein

**Affiliations:** 1 Department of Internal Medicine, Washington University School of Medicine, St Louis, MO 63110 USA; 2 Department of Pathology and Immunology, Washington University School of Medicine, St Louis, MO 63110 USA; 3 Department of Anatomy and Neurobiology, Washington University School of Medicine, St Louis, MO 63110 USA; 4 Department of Pediatrics, Washington University School of Medicine, St Louis, MO 63110 USA; 5 Division of Infectious Diseases, Washington University School of Medicine, 660 S. Euclid Ave., Campus Box 8051, St Louis, MO 63110-1093 USA

## Abstract

Multiple sclerosis (MS) is characterized by episodes of inflammatory demyelination with progressive failure of remyelination. Prior studies using murine models of MS indicate that remyelination within the adult central nervous system (CNS) requires the expression and activity of TNFR2 and CXCR4 by oligodendrocyte progenitor cells (OPCs), promoting their proliferation and differentiation into mature oligodendrocytes. Here, we extend these studies by examining the role of TNFR2 in the expression of the CXCR4 ligand, CXCL12, within the corpus callosum (CC) during cuprizone (CPZ) intoxication and by demonstrating that lentiviral-mediated gene delivery of CXCL12 to the demyelinated CC improves OPC proliferation and myelin expression during remyelination. Activated astrocytes and microglia express both TNFR1 and TNFR2 within the demyelinated CC. However, CPZ intoxicated TNFR2−/− mice exhibit loss of up-regulation of CXCL12 in astrocytes with concomitant decreases in numbers of CXCR4+ NG2+ OPCs within the CC. While CXCR4 antagonism does not affect OPC migration from subventricular zones into the CC, it decreases their proliferation and differentiation within the CC. Stereotactic delivery of lentivirus expressing CXCL12 protein into the CC of acutely demyelinated TNFR2−/− mice increases OPC proliferation and expression of myelin. In contrast, chronically demyelinated wild-type mice, which exhibit significant loss of astrocytes and OPCs, are unable to be rescued via CXCL12 lentivirus alone but instead required engraftment of CXCL12-expressing astrocytes for increased myelin expression. Our results show that TNFR2 activation induces CXCL12 expression in the demyelinated CC via autocrine signaling specifically within astrocytes, which promotes OPC proliferation and differentiation. In addition, gene delivery of critical pro-myelinating proteins might be a feasible approach for the treatment of remyelination failure in MS.

**Electronic supplementary material:**

The online version of this article (doi:10.1007/s00401-012-1034-0) contains supplementary material, which is available to authorized users.

## Introduction

Multiple sclerosis (MS) is an inflammatory, demyelinating disease of the central nervous system (CNS) that presents most often in a relapsing/remitting form, in which a period of demyelination is followed by a period of functional recovery [8, 56]. The recovery stage involves the generation of new myelinating oligodendrocytes, but as the disease worsens, patients exhibit less recovery and accumulate neurological deficits leading to progressive disability. Neurologic functional impairment has been correlated with axonal injury and degeneration, which may occur as a result of remyelination failure or the direct effects of inflammation. While inflammation and remyelination are distinct processes, they are each regulated by master cytokines, such as tumor necrosis factor (TNF-α), that differentially impact infiltrating immune cells and repair of myelin injury. Thus, while successful targeting of TNF-α has led to effective treatments for peripheral autoimmune diseases such as rheumatoid arthritis, psoriasis and inflammatory bowel disease [24, 27, 40], targeting TNF-α in MS patients via administration of a recombinant TNF receptor p55 immunoglobulin fusion protein (lenercept) increased frequency, duration, and severity of MS exacerbations [21], revealing critical roles for this molecule in recovery.

TNF-α is a pleiotropic cytokine that signals through TNFR1 and TNFR2, inducing apoptotic (TNFR1) and survival pathways (TNFR1 and TNFR2) [4, 40]. MS patients exhibit high levels of TNF-α in cerebrospinal fluid and serum, which are positively correlated with lesion severity [23, 31]. In addition, administration of anti-TNF-α agents or use of mice with targeted deletion of TNFR1 in the murine model of MS, experimental autoimmune encephalitis (EAE), revealed a critical role for this receptor in induction of inflammation [17]. Studies examining the roles of TNF-α receptors in remyelination using a model of demyelination within the adult corpus callosum (CC) induced by the copper chelator cuprizone (CPZ), showed that TNFR2 activity is critical for the proliferation and differentiation of OPCs, resulting in attenuation of remyelination after cessation of CPZ [2]. These data suggest that TNFR2 plays a role in CNS repair of myelin and explain the failure of anti-TNF-α agents to alleviate disease in MS patients.

While the mechanism of TNF-α-mediated remyelination is not known, numerous studies indicate that it induces the expression of the chemokine CXCL12 in various cell types within the CNS [1, 22, 25]. CXCL12 is a pivotal regulator of both leukocyte CNS entry and oligodendrocyte differentiation in the adult CNS [36, 37, 43]. CXCL12 is expressed along vascular ablumina and by activated astrocytes and microglia, leading, respectively, to the perivascular localization of infiltrating leukocytes and to the recruitment and maturation of oligodendrocyte progenitor cells (OPCs), which both express the CXCL12 receptor, CXCR4 [36, 37, 43]. Demyelination in the context of CPZ exposure led to increased expression of CXCL12 within the CC. Additionally, loss of CXCR4 signaling, via pharmacological antagonism or RNA silencing, prevented the maturation of OPCs at this site [43]. In addition, prolonged exposure to CPZ, which leads to chronic demyelination, was associated with lack of CXCL12 up-regulation within the CC [43]. These data led us to hypothesize that remyelination failure in TNFR2-deficient mice was similarly linked to loss of CXCL12 expression. Here we report that TNFR2-deficient mice exhibit lack of up-regulation of CXCL12 within activated astrocytes and lower numbers of CXCR4+ OPCs within the CC during CPZ-induced demyelination. BrDU-labeling studies revealed a role for CXCR4 in the proliferation of NG2+ OPCs within the demyelinated CC. Stereotactic delivery of lentivirus expressing CXCL12 into the CC of acutely demyelinated, TNFR2-deficient mice promoted OPC proliferation and myelin expression. In contrast, mice with chronically demyelinated CC due to prolonged CPZ exposure, which display reduced up-regulation of CXCL12 and decreased numbers of activated astrocytes and OPCs, exhibit lack of remyelination rescue in response to lentiviral delivery of CXCL12. These data identify a critical role for TNFR2+ astrocytes in CXCL12-mediated remyelination and demonstrate that gene delivery approaches may ameliorate failed remyelination.

## Materials and methods

### Animals

TNFR1-/-, TNFR2-/- and C57BL/6 control mice were purchased from Jackson Laboratories (Bar Harbor, ME). Breeder colonies of TNFR1-/-, TNFR2-/-, TNFR1,2-/- were maintained at Washington University. All animal procedures were conducted in compliance with NIH guidelines for the use of laboratory animals and were approved by the Animal Studies Committee of Washington University.

### Mouse model of cuprizone-induced demyelination

8- to 10-week old adult male C57BL/6 and TNFR2-/- mice were fed ad libitum 0.2 % CPZ (Harlan Teklan, Madison, WI) mixed in standard rodent chow for 4-12 weeks to induce demyelination of the CC. CPZ-supplemented feed was removed and mice were fed a standard chow diet to induce remyelination [43].

### Antibodies

The following antibodies were used in this study: CXCL12 rabbit polyclonal (eBioscience, San Diego, CA); CXCR4 rat polyclonal (eBioscience); GFAP rat monoclonal (Invitrogen, Carlsbad, CA); IBA1 goat polyclonal (Abcam, Cambridge, MA); MBP rat monoclonal (Abcam); TNFR1 rabbit polyclonal (Abcam); TNFR2 goat polyclonal (R&D systems, Minneapolils, MN); MOG rat monoclonal (R&D systems); BRDU mouse monoclonal (Sigma, St Louis, MO); NG2 rabbit polyclonal (Millipore, Billerica, MA), PDGFRα rat monoclonal (Millipore) and rabbit dsRed for detection of mCherry (Biovision, Milpitas, CA).

### Immunohistochemistry

Mice were intracardially perfused with 4 % paraformaldehyde (PFA) prior to harvest of the brain, which was post fixed overnight in 4 % PFA, cryoprotected in 20 % sucrose, and embedded in frozen medium for sectioning. For immunodetection of chemokine, chemokine and cytokine receptors, cell markers and mCherry, tissue sections were blocked with appropriate concentrations of goat or donkey serum and triton x and then exposed to primary antibody overnight at 4 °C. Slides were washed in PBS followed by incubation of secondary antibodies for 60 min at room temperature. After washing in PBS, slides were nuclear stained with ToPro-3 (Invitrogen) and coverslip were applied with Prolong Gold (Invitrogen) before being visualized on the Zeiss LSM 510 META Confocal Laser Scanning Microscope (Carl Zeiss International, Jena, Germany). Measurement of positive immunofluorescent signals was accomplished using Volocity (Improvision Waltham, MA, USA) or the public domain NIH Image program ImageJ.

### Image analysis

When comparing different treatments (PBS versus AMD3100 or empty vector versus CXCL12 expressing lentivirus) and genotypes (wild-type versus TNFR2-deficient mice) all images were taken at the same exposure settings. To determine the area of double positive labeling, threshold parameters were determined using both the negative controls (isotype control) and images with the lowest fluorescent intensity in both the red and green channels to set the lower threshold setting. Upper threshold settings were not set. The Volocity program was used to determine the area of double labeling and ImageJ was used to determine intensity of signal.

### In vivo AMD3100 treatment

AMD3100 treatment was performed as previously published [36]. Briefly, 28-day osmotic pumps with an infusion rate of 0.11 μl/h (Alzet), containing AMD3100 (40 mg/ml) or vehicle (PBS), were implanted subcutaneously into the flanks of mice at the start of CPZ ingestion for proliferation studies or after 7 days after CPZ start for recruitment studies.

### In vivo bromodeoxyuridine incorporation

Bromodeoxyuridine (BrDU; Sigma) incorporation experiments were performed as previously described [43]. Briefly, BrDU (50–100 mg/kg) was injected into the peritoneal cavity of experimental mice every 8 h for 4 days. For baseline levels of dividing OPCs and for OPC proliferation experiments, mice were injected in this fashion for 4 days prior to killing whereas OPC migration experiments required killing 3 weeks after BrDU exposure.

### Lentivirus

All lentiviruses were produced by the Viral Vectors Core Facility of the Hope Center for Neurological Diseases at Washington University School of Medicine. Murine CXCL12 lentivirus vector (FUW-C) and a second vector encoding mCherry protein alone were generated as previously described [50].

### In vivo lentiviral delivery

Delivery of CXCL12 mCherry versus mCherry expressing lentivirus to the CC was accomplished as previously described [43]. Briefly, mice (groups of 3–5) were anesthetized, positioned in a stereotaxic apparatus and a small incision was made to the scalp [43]. The lateral CC was marked using the following coordinates relative to Bregma: anterior–posterior −2, medial–lateral +0.5, and dorsal–ventral −1.2 [44]. Two microliters of lentivirus were injected using a nanoinjector pump (Stoelting, Wood Dale, IL) into the mouse at a rate of 0.2 μl per min. The mice continued on CPZ feed for 5 days after injection to ensure viral infection. Mice were later switched to standard chow for 10 days before evaluation of CNS tissue.

### In vivo engraftment

Postnatal astroblasts were cultured from 3-day-old wild-type mouse pups as previously described [43]. After 4 days in culture, the cells were infected with either the CXCL12 mCherry versus mCherry expressing lentivirus for 3 days. The cells were enzymatically detached and 60,000 cells were injected into the CC at the same coordinates as the lentivirus injection experiments. The mice continued on CPZ feed for 5 days after injection to ensure viral infection. Mice were later switched to standard chow for 10 days before evaluation of CNS tissue.

### Statistical analyses

Data were analyzed using Prism software (GraphPad Software). The Student’s *t* test was used to determine the statistical significance of immunohistochemistry data. *P* < 0.05 was considered to be significant.

## Results

### TNFR2 mediates expression of CXCL12 within activated astrocytes during CPZ-induced demyelination

**Fig. 1:**
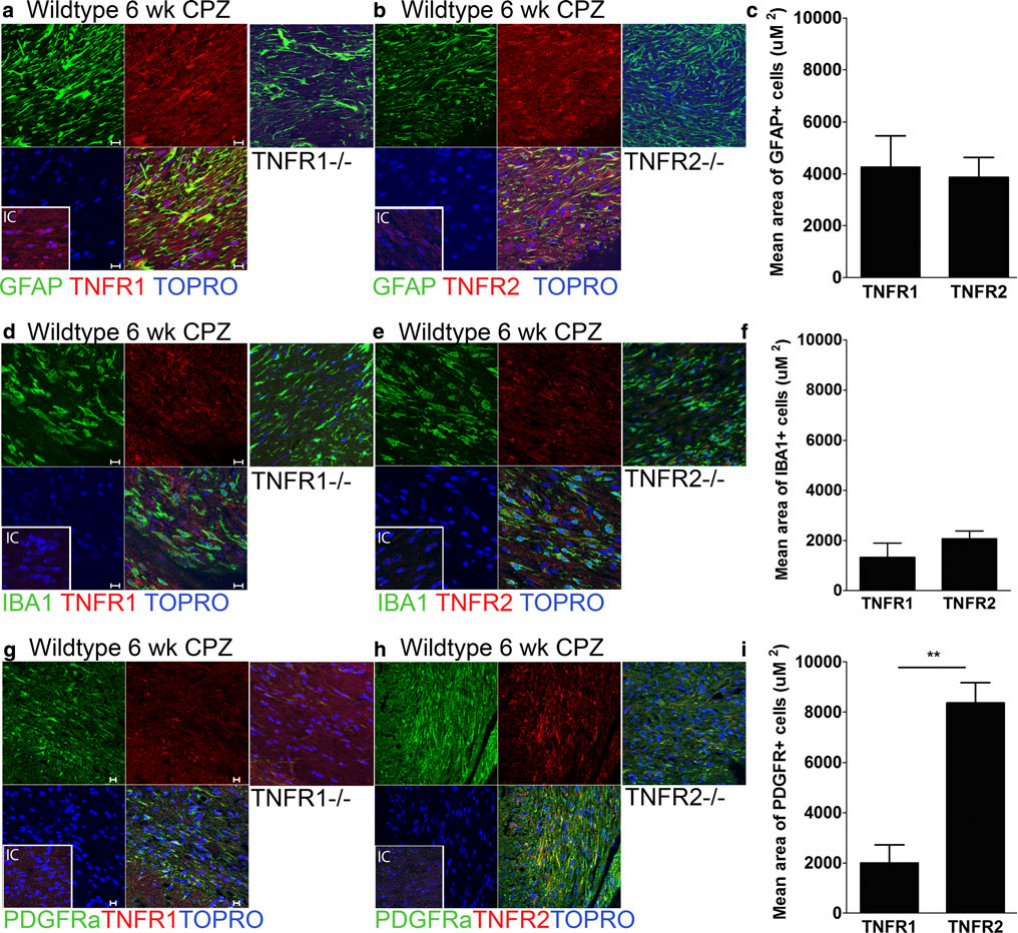
Target cells for TNF-α in demyelinating CC. Wild-type mice were fed a diet containing 0.2 % cuprizone for 6 weeks and then perfused with 4 % PFA. Sections of the CC were stained for GFAP (*green*) (**a**, **b**), IBA1 (*green*) (**d**, **e**), PDGFRα (*green*) (**g**, **h**), and TNFR1 (*left, red*) and TNFR2 (*right, red*). Nuclei were counterstained with ToPro (*blue*). TNFR1−/− (*left*) and TNFR2−/− (*right*) mice after 6 weeks of CPZ ingestion were labeled as negative controls. Images (*n* = 6–8) were taken of three coronal sections from 3 to 5 mice/group using quantitative confocal IHC over two independent experiments. Representative images are shown, *IC* isotype control. *Scale bar* 10 μm. (**c**, **f**, and **i**) The mean area of GFAP + (**c**), IBA1+ (**f**), and PDGFRα+ (**i**) cells that express TNFR1 or TNFR2 within the CC in wild-type mice after 6 weeks of CPZ exposure was quantified. **P < 0.01

**Fig. 2:**
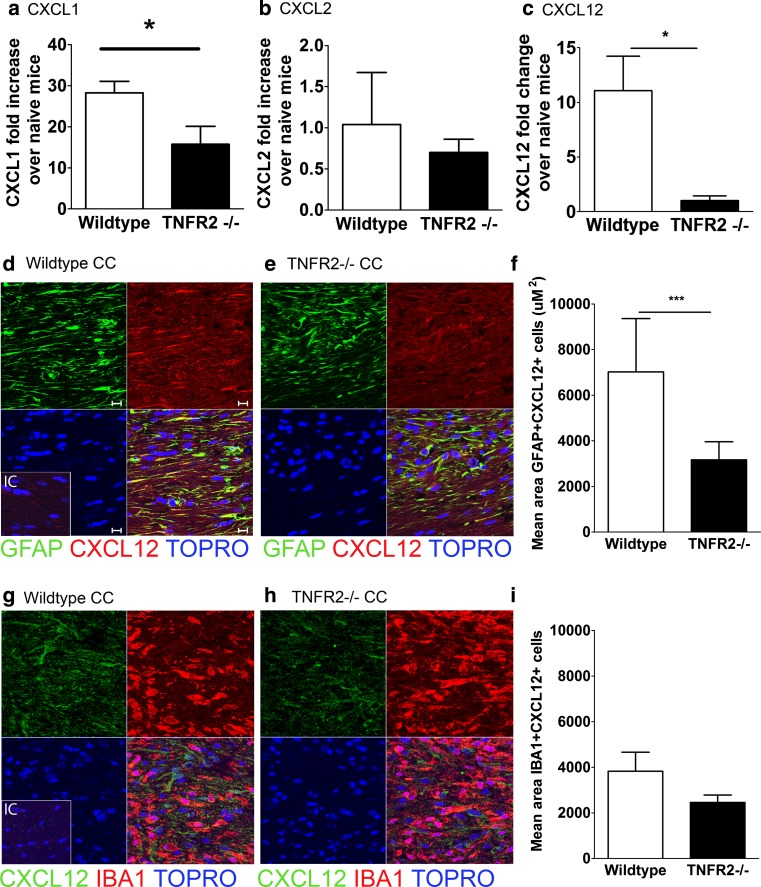
Down-regulated CXCL12 expression in the demyelinated CC of mice lacking TNFR2. Wild-type and TNFR2−/− mice were fed a 0.2 % CPZ-infused diet for 6 weeks. Whole CC was dissected from wild-type and TNFR2−/− mice and qRT-PCR analysis of **a** CXCL1, **b** CXCL2, and **c** CXCL12 expression was performed (*n* = 5 mice/group). Confocal IHC analysis of CC from wild-type and TNFR2−/− mice after 6 weeks of CPZ ingestion. Sections of the CC from (**d**, **g**) wild-type and (**e**, **h**) TNFR2−/− mice were stained for (**d**, **e**) GFAP (*green*) and CXCL12 (*red*) and (**g**, **h**) CXCL12 (*green*) and IBA1 (*red*). All nuclei were counterstained with ToPro (*blue*). Representative images are shown for three coronal sections from three to five mice in two separate experiments, *IC* isotype control. *Scale bars* 10 μm. The mean area of GFAP+ (**f**) and IBA1+ (**i**) cells that express CXCL12 within the CC in wild-type (*white bars*) and TNFR2−/− mice (*black bars*) after 6 weeks of CPZ exposure was quantified; *n* = 6 images taken from three to five mice/group. *P < 0.05, ***P < 0.001

In published studies, astrocytes and microglia within the demyelinated CC of mice exposed to CPZ exhibited increased expression of TNF-α, and activation of TNFR2 proved essential for remyelination upon cessation of toxin. In order to address the mechanism of TNFR2-mediated remyelination, we first identified cellular targets of TNF-α within the CC of naïve and CPZ-demyelinated wild-type mice. Quantitative confocal IHC detection of TNFR1 and TNFR2 in astrocytes (GFAP), microglia (IBA-1) and OPCs (PDGFRα) within the CC was performed on CNS tissues derived from 8-week-old naïve C57BL/6 mice (Supp Figure 1) and from those exposed to CPZ for 6 weeks (Fig. 1). Quantitative confocal IHC revealed high levels of expression of both TNFR1 and TNFR2 by activated astrocytes while microglia expressed much lower levels of both receptors (Fig. 1). OPCs within the demyelinated CC expressed higher levels of TNFR2 than TNFR1 (Fig. 1) (*P* = 0.008), while neither receptor was expressed by any cell type in the CC of naïve mice (Supp Figure 1). Because TNFR2−/−, and not TNFR1−/−, mice exhibited impaired proliferation and maturation of OPCs in the context of CPZ toxicity [2], we wondered whether these receptors differentially impacted the expression of chemokines previously shown to influence the remyelinating capabilities of OPCs [11, 12, 18, 30, 42, 55]. Dissected CC from CPZ-exposed, wild-type mice exhibited increased expression of CXCL1, CXCL2, and CXCL12 mRNAs compared with naïve controls, while those from TNFR2-deficient mice expressed lower levels of CXCL1 (*P* = 0.042) and CXCL12 (*P* = 0.043) (Fig. 2a–c), as assessed via quantitative RT-PCR (QPCR). Quantitative confocal analysis of CXCL12 protein expression within GFAP+ cells of the caudal CC after 6 weeks of CPZ exposure revealed it was significantly decreased in TNFR2−/− mice compared with similarly exposed wild-type animals (Fig. 2d–f) (*P* = 0.011). In contrast, IBA1+ cells within the CC of CPZ-exposed TNFR2-deficient mice exhibited similar levels of CXCL12 expression as wild-type animals (Fig. 2g–i) (*P* = 0.113). Taken altogether, these data indicate that TNFR2 is required for expression of CXCL12 specifically by activated astrocytes within the CC during CPZ-induced demyelination.

### TNFR2-deficient mice exhibit reduced numbers of CXCR4+ OPCs in the CC during CPZ exposure

**Fig. 3:**
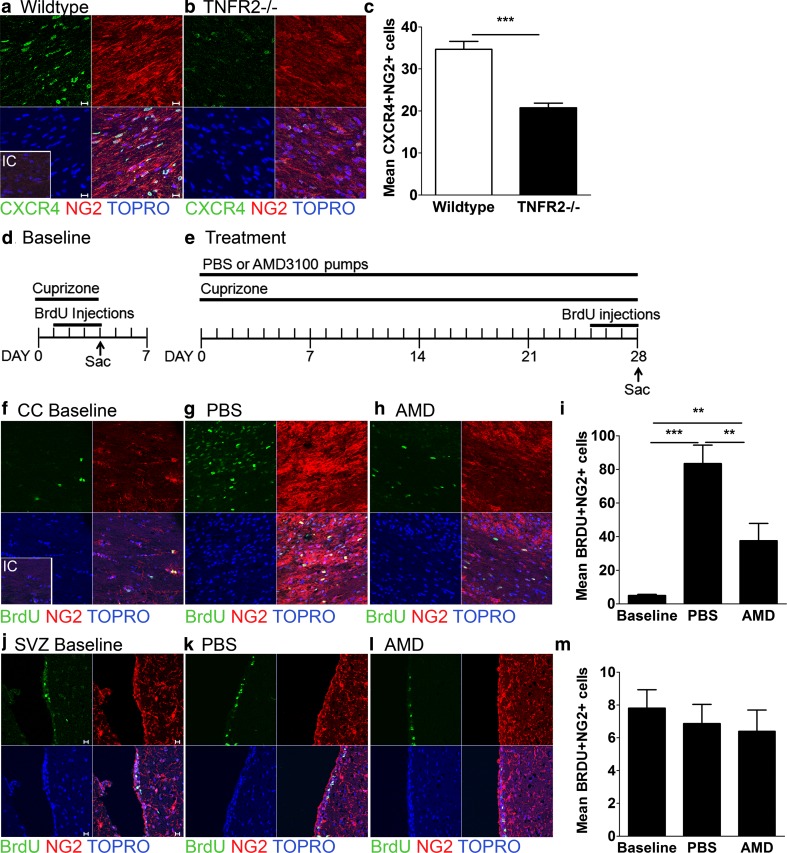
TNFR2 promotes CXCR4-mediated proliferation of OPCs in the demyelinated CC. **a** WT C57Bl/6 and **b** TNFR2−/− mice were fed a diet containing 0.2 % CPZ for 6 weeks and then perfused with 4 % PFA. Cryopreserved sections were stained for CXCR4 (*green*) and NG2 (*red*) Abs. Nuclei were counterstained with Topro (*blue*). **c** The number of CXCR4+ NG2+ cells in the CC was quantified. The depicted timelines represent the experimental design for the **d** baseline and **e** AMD3100- and vehicle-treated mice. Baseline, PBS- and AMD-treated mice were perfused with 4 % PFA and cryopreserved sections of the **f**–**h** SVZ and **j–l** CC were stained for BrDU (*green*) and NG2 (*red*) Abs. Nuclei were counterstained with Topro (*blue*). The mean number of BrDU+ NG2+ cells in the **i** SVZ and **m** CC was quantified

Expression of CXCL12 within the demyelinated, caudal CC has previously been shown to be critical for OPC maturation during remyelination via activation of CXCR4, which is expressed by NG2+ OPCs [43]. These studies, however, did not identify a role for CXCR4 in OPC migration or early proliferation in the context of CPZ toxicity. Because prior studies also determined that TNFR2 activity is required for proliferation of OPCs within the CC during CPZ exposure [2], we hypothesized that impaired remyelination in TNFR2-deficient mice might be due to loss of CXCL12-mediated activation of CXCR4 in OPCs. Indeed, TNFR2−/− mice exposed to CPZ for 6 weeks exhibit significantly decreased numbers of CXCR4+ NG2+ OPCs compared with similarly exposed wild-type controls (Fig. 3a, b) (*P* = 7.83 × 10^−7^). To determine whether CXCR4 activation mediates proliferation of OPCs in the SVZ and the CC during CPZ exposure, we performed BrDU labeling studies in wild-type mice in combination with treatment with the specific CXCR4 antagonist, AMD3100, versus phosphate buffered saline (PBS) vehicle [43]. Baseline BrDU incorporation within OPCs was established by injecting mice with BrDU for four consecutive days beginning at the initiation of CPZ exposure (Fig. 3d). The effect of CXCR4 activation on OPC proliferation was determined via BrDU labeling of OPCs after three weeks of continuous administration of AMD3100 or PBS during CPZ exposure (Fig. 3e). As expected, the CC of mice that were treated with PBS had an expanded population of BrDU+ NG2+ cells, as detected by quantitative confocal IHC, compared to baseline which was significantly decreased in the CC of mice treated with AMD3100 (Fig. 3f–i) (*P* = 0.007). In contrast, proliferation of NG2+ cells in the SVZ was unaffected by CXCR4 antagonism (Fig. 3j–m) (*P* = 0.7884). These data suggest that CXCR4 activation is required for the initial proliferation of OPCs within the demyelinated CC. To determine whether CXCR4-mediated recruitment of OPCs to the CC contributes to the expanded population of NG2+ cells, we used BrDU-labeling to follow OPCs in their migration from the SVZ into the CC in CPZ-exposed mice treated with CXCR4 antagonist. Baseline BrDU labeling was performed in control mice that were exposed to CPZ for 4 days followed by BrDU injections for four additional days while continuing CPZ ingestion (Suppl Figure 2a). AMD3100-or PBS treatment was initiated after BrDU injections and continued along with CPZ exposure for 3 weeks (Supp Figure 2a). Quantitation of total numbers of BrDU+ NG2+ cells within the SVZ and CC prior to AMD3100 or PBS treatment revealed no differences in either brain region (Sup Figure 2i). Taken together, these data indicate that CXCR4 is necessary for OPC proliferation within the demyelinated CC but not for the migration of these cells from the SVZ.

### Delivery of CXCL12-encoding lentivirus rescues remyelination

**Fig. 4:**
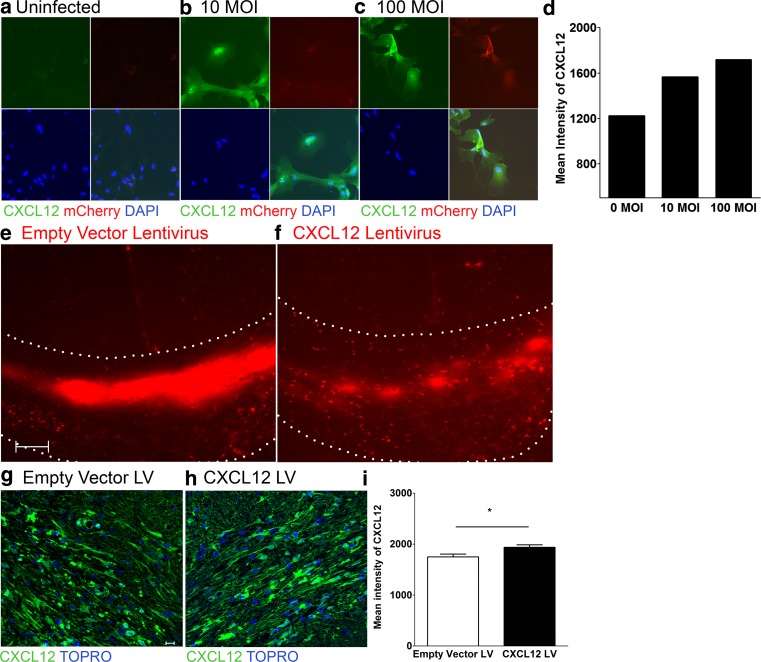
Lentiviral delivery of CXCL12 to the demyelinated CC of TNFR2−/− mice. TNFR2−/− mice were fed a diet containing 0.2 % CPZ for 6 weeks and then a lentivirus containing an empty vector or CXCL12-expressing vector was stereotactically injected into the CC. Mice remained on a CPZ-infused diet for 5 days following injection. The CNS of injected mice was analyzed following a 10-day period on standard chow. **a** Fluorescent IHC analysis of CXCL12 (*green*) and mCherry (*red*) expression by primary astrocytes infected with CXCL12 encoding lentivirus at **a** 0, **b** 10, and **c** 100 MOI. Lower power images depicting stereotactic injection into CC of TNFR2−/− mice with mCherry-expressing lentiviruses encoding **d** empty vector and **e** CXCL12 protein are shown. *Dotted lines* depict the CC. Confocal IHC analysis of CC was performed on lentivirus-injected mice. Images depict **g**, **h** CXCL12 (*green*) expression. Nuclei were counterstained with ToPro (*blue*). Representative images are shown for three coronal sections from three to six mice in two separate experiments. *IC* isotype control. *Scale bars* 10 μm. The mean intensity of CXCL12 expression (**i**), within the CC of TNFR2−/− mice injected with the empty vector (*white bars*) and CXCL12 (*black bars*) lentivirus after 6 weeks of CPZ exposure was quantified; *n* = 6 images were taken from three to six mice/group. *P < 0.05

We and others have shown that CXCL12 is required to promote remyelination of the injured adult CNS [10–12]. Given that TNFR2-deficient mice exhibit lack of CXCL12 up-regulation within astrocytes of the demyelinated CC (Fig. 2f), we wondered whether reinstating CXCL12 expression at this site might rescue the delayed remyelination observed in TNFR2−/− mice. Two lentiviral constructs were used to test this hypothesis, one encoding-CXCL12 and mCherry and another encoding mCherry alone [50]. In vitro infection of astrocytes with lentivirus encoding both CXCL12 and mCherry confirmed transduction and CXCL12 expression as assessed by immunocytochemistry (Fig. 4a–d). After stereotactic injection of either lentivirus into the CC of CPZ-exposed wild-type or TNFR2−/− mice, mCherry-expression could be detected in the CC in either genotype within 10 days (Fig. 4e, f). Quantitative confocal IHC detection of CXCL12 protein, however, revealed it was significantly increased within the CC of mice that received CXCL12-expressing lentivirus compared with those that received empty vector lentivirus (Fig. 4g, h) (*P* = 0.0131).

**Fig. 5:**
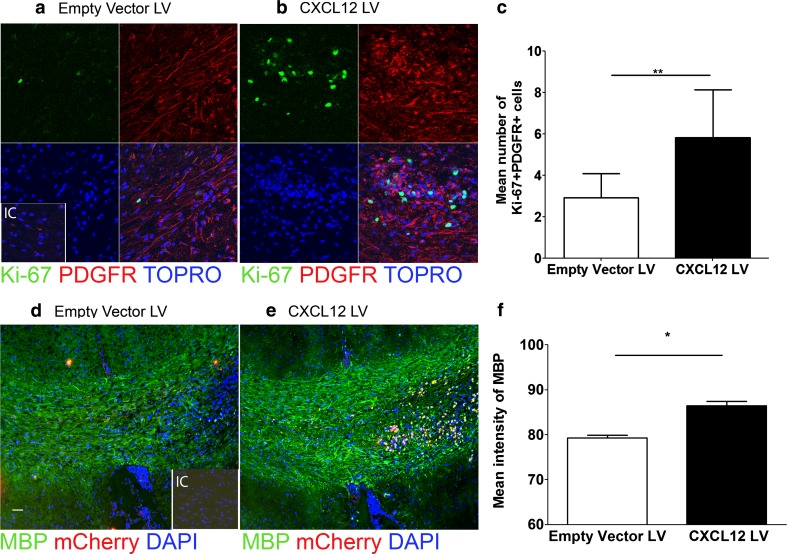
Lentiviral delivery of CXCL12 rescues remyelination in TNFR2−/− mice. The CC of TNFR2−/− mice after 6 weeks of CPZ followed by stereotactic injection with mCherry-expressing lentiviruses encoding empty vector (*left*) and CXCL12 (*right*) protein was analyzed via quantitative confocal IHC. Images depict mCherry expression (*red*) with staining for (**a**, **b**) Ki-67 (*green*) PDGFRα (red) and (**d, e**) MBP (green). Nuclei are counterstained with ToPro or DAPI (*blue*). Representative images shown for three coronal sections from three to six mice in two separate experiments. *IC* isotype control. *Scale bars* 10 μm. The number of PDGFR+ Ki-67+ cells (**c**), and intensity of MBP expression (**f**) within the CC of empty vector (*white bars*) and CXCL12 (*black bars*) lentivirus-infected mice was quantified; *n* = 6 images taken from three to six mice/group. *P < 0.05, **P < 0.01

To determine whether reinstating CXCL12 in the CC of CPZ-exposed, TNFR2-deficient mice would alter oligodendrocyte biology and remyelination, we examined OPC proliferation and myelin protein expression within the CC of lentivirus-infected mice via quantitative confocal IHC. TNFR2-deficient mice that received CXCL12-encoding lentivirus exhibited increased numbers of Ki-67+ PDGFRα+ OPCs (Fig. 5a–c) (*P* = 0.008) compared with those that received empty vector lentivirus. Mice injected with CXCL12-encoding lentivirus also exhibited a significant increase in MBP expression compared with mice injected with nonspecific lentivirus (Fig. 5d–f) (*P* = 0.003). These data indicate that CXCL12 reinstatement can improve remyelination in TNFR2-deficient mice with CPZ-induced demyelination.

**Fig. 6:**
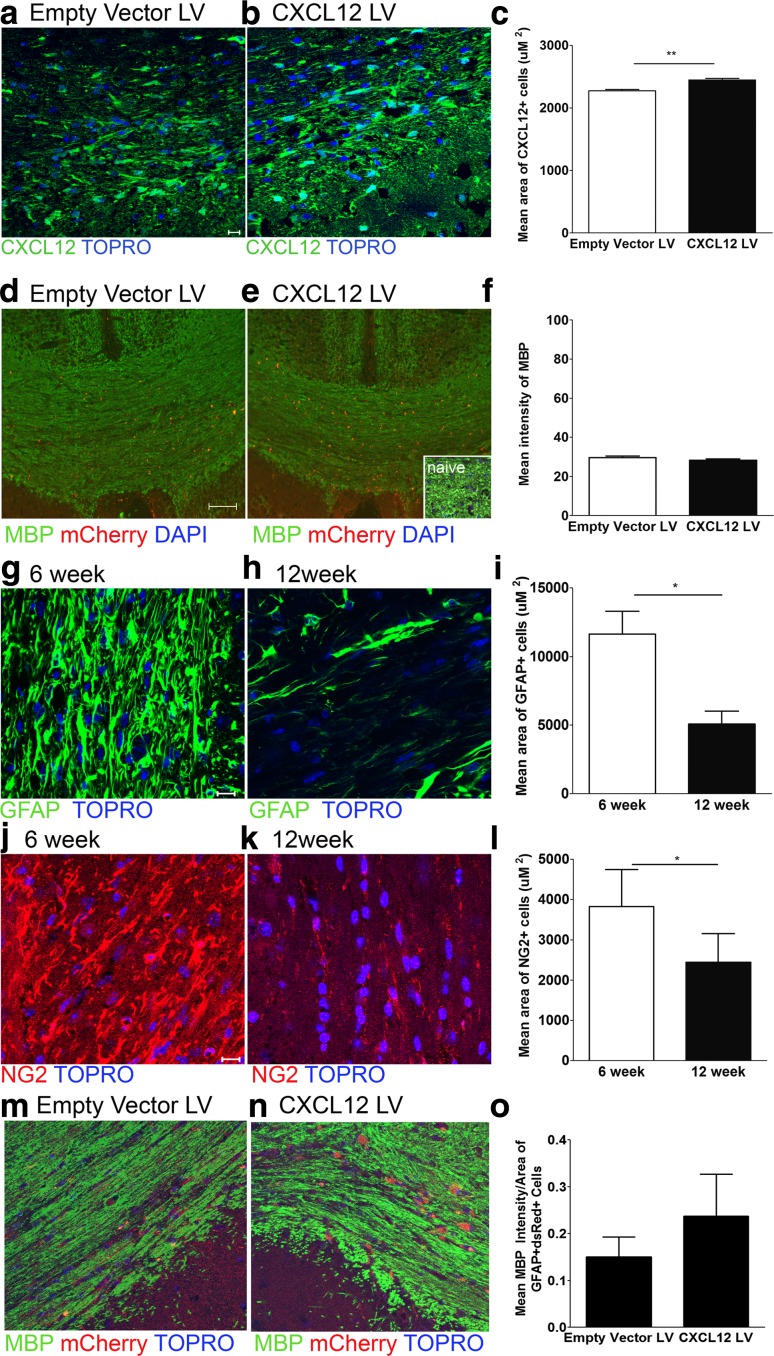
Enhanced CXCL12 expression does not rescue remyelination following chronic demyelination. Wild-type mice were fed a diet containing 0.2 % CPZ for 12 weeks and then the CC were injected with mCherry-expressing lentiviruses encoding empty vector (*left*) and CXCL12 (*right*) protein. Quantitative confocal IHC was used to analyze the CC 10 days after the cessation of CPZ and stereotactic lentiviral injections. Images depict mCherry expression (*red*) with staining for (**a**, **b**) CXCL12 (*green*) and (**d**, **e**) MBP (*green*). Nuclei are counterstained with ToPro (*blue*). *Inset* depicts MBP staining in naïve CC as a positive control. Representative images are shown for three coronal sections from three to six mice in two separate experiments. *IC* isotype control. *Scale bars* 10 μm. The area of CXCL12+ cells (**c**) and intensity of MBP expression (**f**) within the CC of empty vector (*white bars*) and CXCL12 (*black bars*) lentivirus-infected mice was quantified. **P < 0.01. Wild-type mice were fed a diet containing 0.2 % CPZ for 6 weeks (*left*) or 12 weeks (*right*) and the CC were then analyzed using quantitative confocal IHC. Sections of CC were stained for (**g**, **h**) GFAP (*green*) or (**j**, **k**) NG2 (*red*) and nuclei were counterstained with ToPro (*blue*). Representative images are shown for three coronal sections from 4 mice in two separate experiments. *IC* isotype control. *Scale bars* 10 μm. The area of GFAP+ (**i**) or NG2+ (**l**) cells in wild-type mice after 6 (*white bars*) and 12 (*black bars*) weeks of CPZ exposure was quantified, *n* = 6 images taken from 4 mice/group. *P < 0.05. Engraftment of astrocytes infected with CXCL12-encoding lentivirus rescues remyelination following chronic demyelination. Wild-type mice were fed a diet containing 0.2 % CPZ for 12 weeks and then the CC were injected with cultured astrocytes infected with mCherry-expressing lentiviruses encoding empty vector and CXCL12 protein. Quantitative confocal IHC was used to analyze the CC 10 days after the cessation of CPZ and stereotactic injections. Images depict immunodetection of mCherry expression (*red*) with staining for (**m,** **n**) MBP (*green*). Nuclei are counterstained with ToPro (*blue*). The intensity of MBP expression/area of GFAP+ mCherry+ (**o**) within the CC of demyelinated mice (*black bars*) was quantified. Representative images are shown for three coronal sections from 5 mice/group. *Scale bars* 10 μm

Prolonged CPZ exposure beyond 12 weeks leads to chronic demyelination that persists following CPZ cessation [35]. Given that remyelination was rescued in TNFR2-deficient mice after CXCL12 reinstatement, we wondered whether CXCL12 lentivirus might similarly promote remyelination in chronically demyelinated mice. CXCL12-encoding or empty vector lentiviruses were stereotactically injected into the CC of wild-type mice after 12 weeks of CPZ exposure. CXCL12 protein expression at 10 days after both CPZ cessation and lentiviral infection was significantly increased within the CC of chronically demyelinated mice that received CXCL12 compared to those that received empty vector virus (Fig. 6a–c) (*P* = 0.003). Quantitative confocal IHC evaluation of MBP expression in lentivirus-infected CC, however, did not reveal any differences between CXCL12 and empty vector viruses (Fig. 6d–f) (*P* = 0.24). Of interest, GFAP+ astrocytes (*P* = 0.0119) and NG2+ (*P* = 0.0118) cell numbers were significantly decreased in the CC derived from mice exposed to CPZ for 12 weeks when compared with the CC of mice fed CPZ for 6 weeks (Fig. 6g–l), suggesting that lack of astrocyte targets for lentivirus infection or sufficient OPC numbers to respond to CXCL12 expression prevented remyelination rescue.

Given these data, we wondered whether engraftment of CC with astrocytes infected with mCherry and CXCL12-encoding lentivirus would provide the necessary astrocytes to rescue remyelination. Postnatal astroblasts infected with mCherry and CXCL12-encoding or empty vector lentiviruses were stereotactically injected into the CC of wild-type mice after 12 weeks of CPZ exposure. Quantitative confocal IHC evaluation of MBP or MOG expression per area of GFAP+ mCherry+ cells in CC revealed increased expression of MBP (Fig. 6m–o) in the mice engrafted with astrocytes infected with CXCL12-encoding lentivirus versus those receiving astroblasts infected with empty vector virus. These data indicate that engraftment of CXCL12-expressing astrocytes can improve remyelination in the chronically demyelinated mice.

## Discussion

**Fig. 7:**
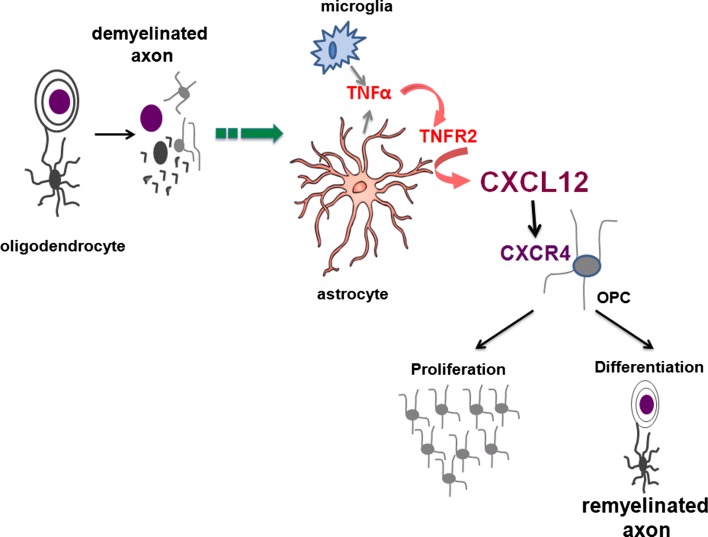
TNF-α-mediated mechanisms of remyelination. TNF-α, TNFR2, CXCL12, CXCR4, astrocytes and OPCs interact in the context of remyelination. In the CPZ model, ingestion of the copper chelator leads to complete demyelination of the CC via apoptosis of oligodendrocytes. This causes the activation of microglia and astrocytes that express both TNFR1 and TNFR2. Microglia and astrocytes produce TNF-α. On astrocytes, the activation of TNFR2 induces CXCL12 expression. In turn, CXCL12 activates CXCR4, which is expressed by OPCs. Activation of CXCR4 causes the proliferation of OPCs and differentiation into myelinating oligodendrocytes

While the exact mechanisms of remyelination in the adult CNS are unknown, numerous studies suggest that astrocyte reactivity is positively correlated with endogenous repair of myelin [7, 16, 38]. Astrocytes respond to inflammatory cytokines by secreting growth factors and chemokines that are known to impact OPC biology [5, 28, 33, 34, 41, 43]. Although it is well-established that TNF-α induces CXCL12 expression, it is not known which receptor is responsible for this induction. TNFR2 signaling activates prosurvival pathways through the recruitment of TRAF2 adapter proteins and subsequent activation of NFκB, which is known to induce chemokine genes [9, 13]. In splenocytes, NFκB p52/RelB heterodimers induce CXCL12 transcription [26]. Consistent with this, our data indicate that activated astrocytes express TNFR2, which promotes OPC proliferation and differentiation via induction of the chemokine CXCL12. We show that mice fed the demyelinating intoxicant CPZ display increased expression of both TNFR1 and TNFR2 by activated astrocytes within the CC. TNFR2−/− mice, however, which exhibit delayed remyelination after CPZ cessation [2], show reduced up-regulation of astrocyte CXCL12 expression within the CC compared with wild-type animals. Accordingly, reduced numbers of CXCR4 + OPCs were observed within the CC of TNFR2-deficient mice during CPZ-induced demyelination. BrDU-labeling studies in mice treated with a CXCR4 antagonist during CPZ exposure revealed decreased proliferation, but not migration, of OPCs within the CC when compared with vehicle-treated controls, suggesting that CXCL12 acts primarily within the demyelinated CC to induce the proliferation of recruited OPCs. In support of this, lentiviral delivery of CXCL12 expression to the CC of CPZ exposed, TNFR2-deficient mice enhanced OPC proliferation and cell numbers, leading to increased expression of myelin compared with mice that received empty vector lentivirus. In contrast, chronically demyelinated CC, which exhibit decreased astrocyte and OPC numbers, do not display remyelination rescue in response to CXCL12 lentivirus. These data indicate that TNF-α expression by the demyelinated CC induces repair via astrocyte expression of chemokines that target OPCs (Fig. 7).

The role of TNF-α in the pathogenesis of CNS autoimmunity is unclear. In studies utilizing the murine MS model, EAE, both TNF-α- and TNFR1-deficient mice are resistant to disease and the use of anti-TNF or sTNF receptor antibodies reduced disease severity [17, 46, 47]. In addition, transgenic mice that over-express TNF-α spontaneously develop chronic, inflammatory demyelinating disease [45]. In human studies, the detection of TNF-α in both the cerebrospinal fluid of MS patients and within MS brain lesions [48] also suggested it might be targeted to ameliorate disease. However, Phase II clinical trials in which patients with relapsing-remitting MS were administered Lenercept, a sTNF-RI fusion protein that neutralizes TNF-α were halted when disease acutely worsened [21]. The ineffectiveness of anti-TNF-α therapy in humans, contrary to animal studies, suggested divergent roles for TNF-α receptors in inflammation and repair. Both TNFR1 and TNFR2 can exhibit neuroprotective effects. For example TNFR1 has been shown to protect neurons against excitotoxicity by sensitizing the neurons to erythropoietin and vascular endothelial growth factor (VEGF) and TNFR1 signaling can also activate NFκB which induces prosurvival genes [32, 51]. Under certain conditions TNFR1 signaling may activate neuroprotective mechanisms. However, the predominant signaling pathway is not neuroprotective but instead leads to apoptosis [4]. In contrast, TNFR2 signaling is predominantly neuroprotective as shown by the use of TNFR2-selective agonist that rescues neurons from oxidative stress-induced cell death [15]. Consistent with this, TNFR2-deficient mice exhibit delayed repair in models of remyelination [2] and targeted deletion of tumor necrosis factor receptor associate-factor 4 (TRAF4), a major signal transducer for TNF-α led to ultrastructure perturbation in myelin [6]. These data suggest that therapeutic strategies that antagonize TNFR1 to limit inflammation and augment TNFR2 signaling to enhance remyelination might provide effective treatments that both ameliorate disease and promote recovery in MS patients.

Our results demonstrated that during CPZ-induced demyelination, OPCs express TNFR2 in greater numbers than TNFR1. However, TNFR2-deficiency results in lower numbers of CXCR4+ OPCs within CC after demyelination, suggesting that TNFR2 signaling impacts OPC biology. We and others previously reported that CXCR4 signaling in OPCs is required for their differentiation into myelinating oligodendrocytes in murine models of demyelination [11, 43]. Studies using the CPZ model of demyelination, however, did not address the role of CXCR4 in the recruitment of OPCs from the SVZ. In our current study, we determined that CXCR4 signaling is required for proliferation of OPCs within the CC during demyelination but not for the migration of OPCs to this site. Thus, the localized demyelination of the CC and expression of CXCL12 by astrocytes initiates proliferation of OPCs via CXCR4 signaling directly at the site of the injury. The migration of OPCs from the SVZ is not directed by CXCL12, but instead maybe directed by other chemokines such as CXCL1 and/or CXCL2. Studies have demonstrated that CXCR2, the receptor for CXCL1 and CXCL2, is expressed by OPCs and oligodendrocytes [29] and has been shown to direct OPC positioning during spinal cord development [52]. TNFα has been shown to induce CXCL1 and CXCL2 in cultured mouse astrocytes and stereotaxic injection of TNF-α to the SJL and BALBc mouse brains induced CXCL2 [18, 42, 55]. Given that we found attenuated CXCL1 message in the TNFR2 deficient mice and decreased numbers of OPCs within the CC, these data suggest that TNFR2 signaling may mediate migration of OPCs via CXCL1 signaling. However, further experiments are necessary to determine the individual contributions of TNFα/TNFR2, CXCL12/CXCR4, and CXCL1/CXCR2 in regulating remyelination.

Other published studies have reported that CXCR4 plays an important role in all aspects of oligodendrocyte biology, including migration. Cultured neonatal OPCs respond to CXCL12 via directed chemotaxis and both CXCR4 and CXCR7, a non-signaling CXCL12 receptor, influence OPC maturation [14, 19]. In in vivo studies, however, Carbajal et al. [10] reported that CXCR4, but not CXCR7, plays a role in the migration and differentiation of OPCs in a model of chronic demyelination within the spinal cord after primary infection with mouse hepatitis virus (MHV) [11]. Furthermore, in follow-up studies, CXCR4 antagonism was shown to inhibit endogenous OPC maturation and increased the rate of proliferation in the MHV viral model [10]. In the EAE model, Olig2-EGFP+ or CXCR4-EGFP+ oligodendrocyte progenitors implanted into the lateral ventricles of mice at peak of disease migrated into the CC in response to elevated CXCL12 levels and differentiated into mature oligodendrocytes [3]. In addition, migration was inhibited after RNA silencing of CXCR4 [3]. Taken together these reports demonstrate that CXCL12/CXCR4 are important players in mediating OPC biology and an alteration in expression levels of CXCL12 or CXCR4 influences remyelination.

Our results show that TNFR2 deficient mice have reduced CXCL12 expression by astrocytes after CPZ-induced demyelination and that this correlates with reduced OPC numbers. Under normal conditions, astrocytes are a major cellular component of the CNS that provide metabolic and trophic support to maintain homeostasis [49, 53], however, the role of astrogliosis in CNS repair remains controversial. The presence of reactive astrocytes in areas of demyelination has been observed to decrease remyelination within the spinal cord after transplantation of OPCs [7]. In addition, astrocytes isolated from contused spinal cord inhibited the in vitro differentiation of OPCs, whereas astrocytes derived from normal spinal cord did not [54]. However, in several demyelinating models, including cuprizone and EAE, proliferating astrocytes and their secreted factors can promote remyelination in both the spinal cord and CC [16, 38, 39, 43]. Consistent with this, expression of CXCL12 by activated astrocytes within the acutely demyelinated CC influences remyelination by mediating the proliferation and differentiation of OPCs. Reduced astrocyte activation or numbers in the chronically demyelinated CC might therefore negatively influence OPC differentiation and functional myelin formation through loss of a proliferative or trophic microenvironment.

Alterations in astrocyte activity may also have an impact on the success of treatments that attempt to augment or enhance remyelination in MS patients. Lentiviral vectors, for example, are able to preferentially transduce major glial cell types within white matter, such as astrocytes [57]. In our study, CXCL12-encoding lentivirus promoted OPC proliferation and remyelination within the demyelinated CC of TNFR2-deficient mice, which exhibit large numbers of activated astrocytes, but not in the CC of chronically demyelinated mice, which exhibit astrocyte depletion. In addition, as OPC numbers are also reduced during prolonged CPZ treatment, reinstating CXCL12 may exert little effect on their proliferative capacity and ultimate ability to remyelinate. However, further studies are necessary since restoration of depleted OPC and astrocyte populations may require more time to show sufficient remyelination after chronic demyelination. Given the role of CXCL1 on OPC recruitment, lentiviruses expressing multiple chemokines might be required to promote OPC recruitment, proliferation and differentiation within MS lesions. In addition, ex vivo gene therapy, in which cells are genetically modified prior to implantation into the brain, might be employed to replenish depleted astrocytes that augment repair signals [20]. In our study, CXCL12-expressing astrocytes engrafted into the CC of chronically demyelinated showed an increase in myelin expression suggesting that remyelination may be rescued with chemokines and cells that influence chemokine expression in the brain. Such gene therapy or stem cell engraftment approaches may enable the localized manipulation of factors to induce remyelination within various types of MS lesions.

In summary, our data indicate that TNFR2 signaling by activated astrocytes within the demyelinated adult CNS is essential for their expression of CXCL12, which facilitates OPC proliferation and myelin repair. Lentiviral vector-based gene delivery of CXCL12 improved remyelination specifically where loss of astrocytes or OPCs did not prevent lentiviral or CXCL12 targeting. These studies enhance our understanding of cytokine-mediated recovery from demyelination and demonstrate that reinstating chemokine expression may be a feasible approach for the treatment of MS.

## Electronic supplementary material

Below is the link to the electronic supplementary material.

Supplementary material 1 (DOC 11 kb)

Supplementary material 2 (TIFF 49877 kb)

Supplementary material 3 (TIFF 50790 kb)
