# Supplementary material for: Astrocyte TNFR2 is required for CXCL12-mediated regulation of oligodendrocyte progenitor proliferation and differentiation within the adult CNS
Source: Acta Neuropathol. 2012 Aug 30;124(6):847–60. doi: 10.1007/s00401-012-1034-0 (PMC3508279; doi:10.1007/s00401-012-1034-0)
Supplement: Supplementary file 1 — Supplementary material 1 (DOC 11 kb) [file 401_2012_1034_MOESM1_ESM.doc]

**Supplemental Figure 1.** Naïve mice do not express TNFR1 and TNFR2. Naïve Wild-type, TNFR1-/-, TNFR2-/- and TNFR1/2-/- were stained for MBP (green) and **(a)** TNFR1 or **(b)** TNFR2 (red) and nuclei were stained with ToPro (blue). Quantitative confocal IHC was used to determine receptor expression in the CC. Representative images are shown for three coronal sections from three to five mice. IC = isotype control. *Scale bars*, 10 µm.

**Supplemental Fig.2** CXCR4 does not impact the migration of OPCs to the demyelinated CC. Male C57Bl/6 mice were fed a CPZ-infused diet for 4 days and then injected with BrDU for 4 consecutive days. Baseline mice were perfused with 4% PFA 6 h following the final BrDU injection. AMD3100- and PBS-treated mice were implanted with osmotic pumps 6 h following the final BrDU injection and perfused with 4% PFA 3 weeks later. (**a**) The depicted timeline represents the experimental design. Cryopreserved sections of the (**b**-**d**) SVZ and (**e**-**g**) CC were stained for BrDU (*green*), NG2 (*red*), and Topro (*blue*). The mean number of BrDU+NG2+ cells in the (**d**) SVZ and (**g**) CC was quantified.
