# Supplementary figures and images for: Astrocyte TNFR2 is required for CXCL12-mediated regulation of oligodendrocyte progenitor proliferation and differentiation within the adult CNS
Source: Acta Neuropathol. 2012 Aug 30;124(6):847–60. doi: 10.1007/s00401-012-1034-0 (PMC3508279; doi:10.1007/s00401-012-1034-0)

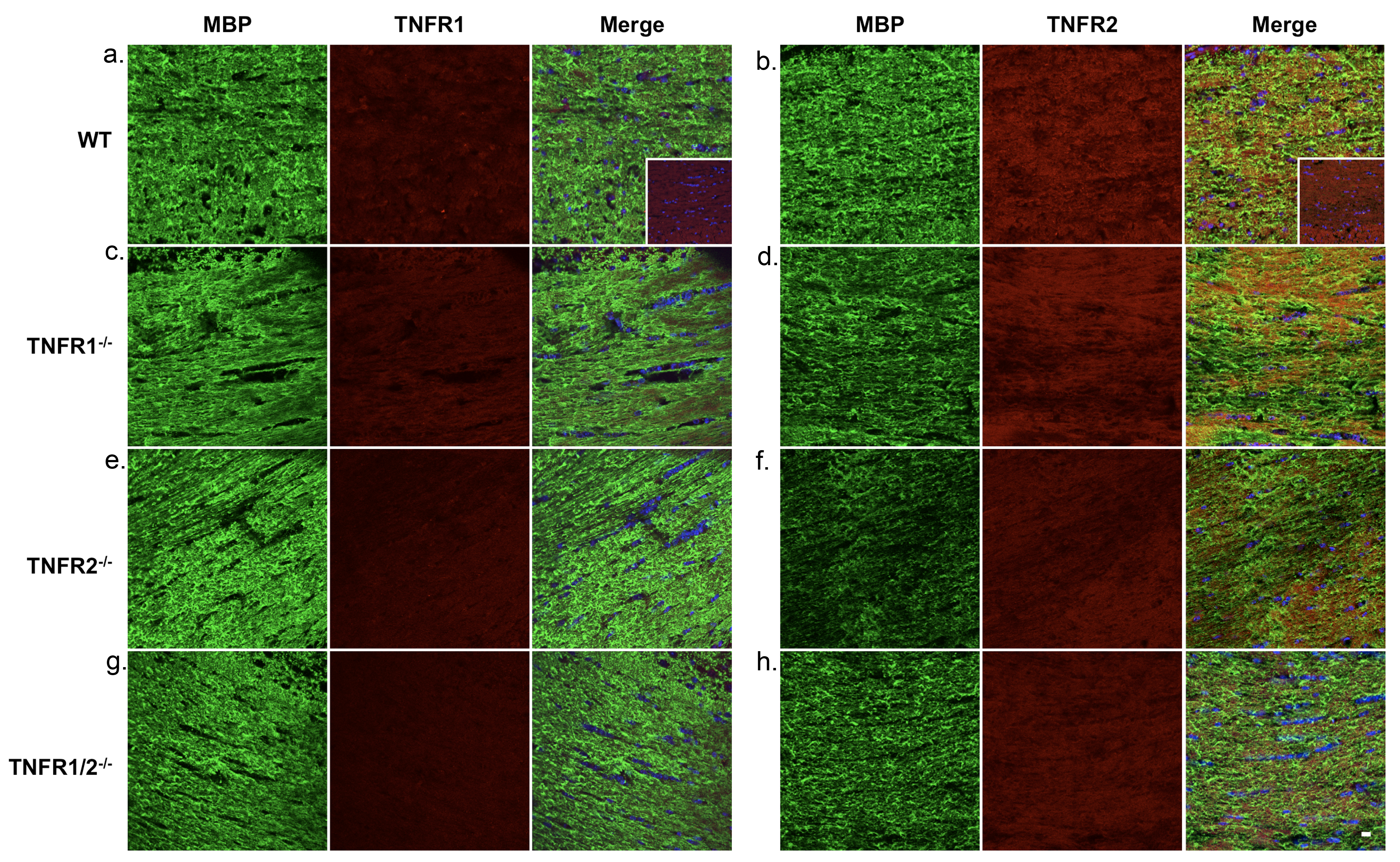

Supplement: Supplementary file 2 — Supplementary material 2 (TIFF 49877 kb) [file 401_2012_1034_MOESM2_ESM.tif]

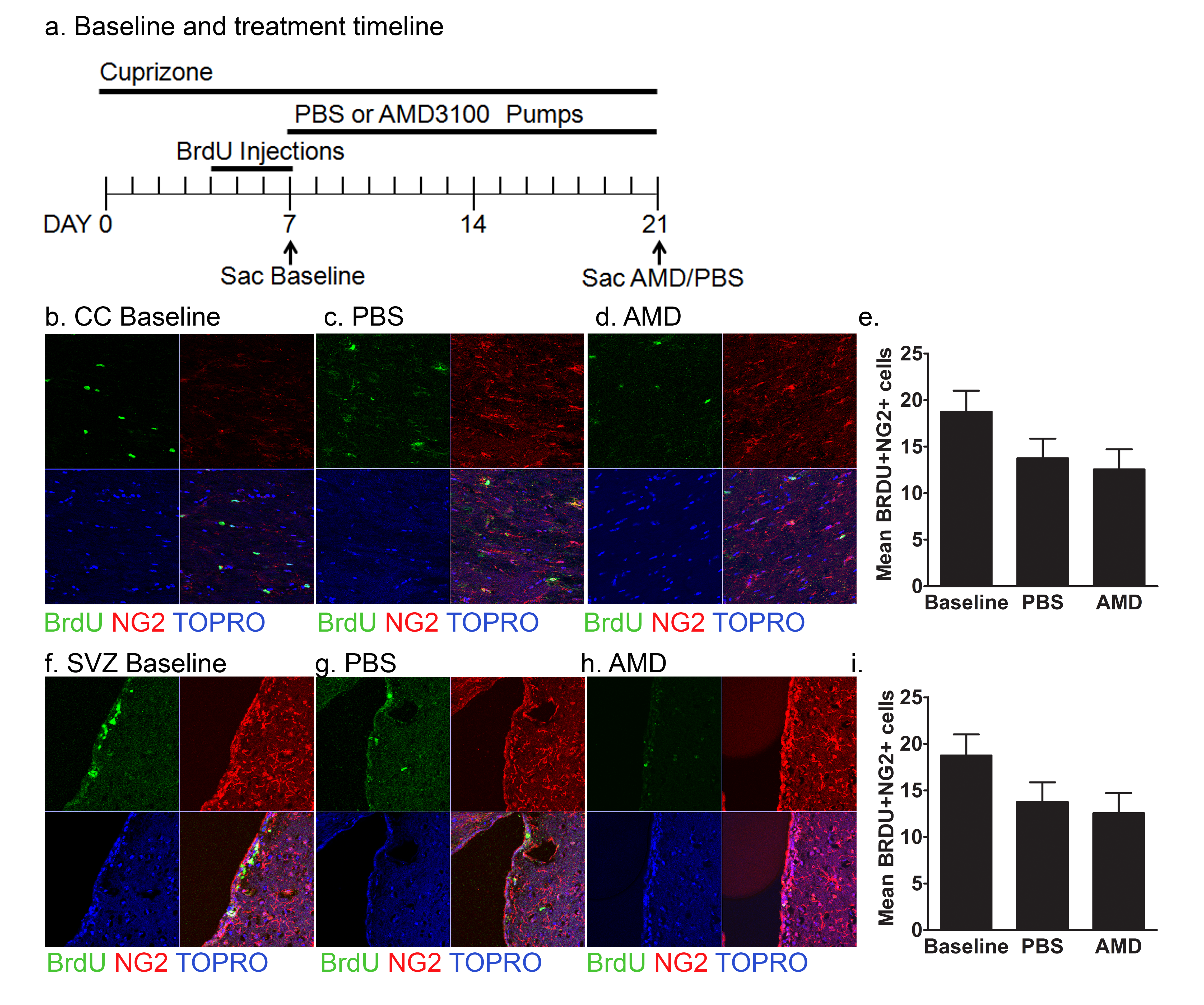

Supplement: Supplementary file 3 — Supplementary material 3 (TIFF 50790 kb) [file 401_2012_1034_MOESM3_ESM.tif]
